# Portable High Voltage Integrated Harvesting-Storage Device Employing Dye-Sensitized Solar Module and All-Solid-State Electrochemical Double Layer Capacitor

**DOI:** 10.3389/fchem.2018.00443

**Published:** 2018-09-25

**Authors:** Alberto Scalia, Alberto Varzi, Andrea Lamberti, Timo Jacob, Stefano Passerini

**Affiliations:** ^1^Helmholtz Institute Ulm (HIU), Ulm, Germany; ^2^Karlsruhe Institute of Technology (KIT), Karlsruhe, Germany; ^3^Department of Applied Science and Technology, Politecnico di Torino, Turin, Italy; ^4^Institute of Electrochemistry, Ulm University, Ulm, Germany

**Keywords:** supercapacitor, dye sensitized solar cell, integrated device, portable device, polymer electrolyte, harvesting-storage device

## Abstract

A dye-sensitized solar module (DSSM) and a high voltage all-solid-state electrochemical double layer capacitor (EDLC) are, for the first time, implemented in a compact Harvesting-Storage (HS) device. Conductive glass is employed as current collecting substrate for both DSSM and EDLC, leading to a robust and portable final structure. The photovoltaic section is constituted by a 4 series cells W-type module, while in the storage section an EDLC employing an ionic liquid-based polymeric electrolyte (a mixture of polyethylene oxide and N-butyl-N-methylpyrrolidinium bis(trifluoromethanesulfonyl)imide, PEO-Pyr_14_TFSI) and activated carbon electrodes is used. The solid state EDLC is first characterized individually to determine its electrochemical performance before successfully proving the integration with the DSSM. The harvesting-storage properties of the integrated photo-capacitor are evaluated through photo-charge and subsequent discharge protocols performed at two different discharge currents, showing that in this configuration the EDLC unit can be effectively charged up to 2.45 V.

## Introduction

Fossil fuels are the worldwide most employed energy source. In the past, their relative abundance has promoted their use in several contexts: automotive, residential heating, industry, power plants, etc. However, the increasing level of pollution, poor air quality and global warming caused by their excessive employment is forcing the international community to shift toward greener and renewable alternatives, such as photovoltaic (PV) power generation (Gonçalves et al., 2008; El Chaar et al., 2011; Parida et al., 2011). In the emerging PV technology framework, different technologies have been proposed. Among these, dye-sensitized solar cells (DSSCs) possess the lowest fabrication cost and the smallest payback time (Wongcharee et al., 2007; Zardetto et al., 2013; Liu et al., 2015a,b; He et al., 2017; Hwang et al., 2017; Li et al., 2017). In addition, after years of research they have reached appreciable efficiency and reliability (Jung and Lee, 2013; Gerosa et al., 2016).

However, the intermittent nature of the incident solar radiation makes an uninterrupted power supply impossible. Thus, for many applications, a storage media able to accumulate the converted solar radiation is mandatory in order to stabilize the electrical output of the PV section and, therefore, the input for the user (Ng et al., 2015; Luo et al., 2017). Recently, the idea to directly connect the PV harvester with a storage unit in a monolithic integrated device is widely considered (Schmidt et al., 2016). This integration is needed to drive users, such as electronic devices or sensors, (Volkov et al., 2017; Yang et al., 2017; Garcia-Hernandez et al., 2018; Sorvin et al., 2018) particularly when the access to the electric grid is denied. Electrochemical double layer capacitors (EDLCs) are particularly suitable for integration with PV technology. This is mainly due to their outstanding cycling stability. In fact, EDLCs can sustain a rather high number of charge–discharge cycles (even above 1,000,000) (Zhang and Pan, 2015) without an appreciable change of their capacitance nominal value. To date, batteries have not shown the same longevity (Zhang and Pan, 2015). Furthermore, they are less sensitive to the voltage output of the harvesting section than batteries, which, depending on the chemistry, require a well-defined operative voltage to be charged. Last, EDLCs can withstand wide current ranges being definitely more versatile (Choudhury et al., 2009; Zhong et al., 2015; González et al., 2016; Lin et al., 2018).

Most of the integrated devices presented in literature(Wei et al., 2017; Yun et al., 2018) deal with a single solar cell connected to an electrochemical double layer capacitor (EDLC) or a battery (Xu et al., 2014; Kim et al., 2017; Scalia et al., 2017). Thus, the final voltage obtained in the storage section is generally lower than 1 V (Chen et al., 2012; Skunik-Nuckowska et al., 2013; Yang et al., 2013; Cohn et al., 2015; Li et al., 2016). Recently, however, the feasibility of photocapacitors with a harvesting section composed by more solar cells in series has been demonstrated. In a previous work, (Scalia et al., 2018) a voltage well beyond 2 V was obtained using a highly stable ionic liquid electrolyte in the storage section, thus guaranteeing an impressive discharge capacity of 0.1 mAh·cm^−2^ subsequent to photo-charge. The N-butyl-N-methylpyrrolidinium bis(trifluoromethanesulfonyl)imide (Pyr_14_TFSI) ionic liquid demonstrated stability during charge–discharge protocols lasting for several hours. In addition, the discharge capacity measured after 1 h rest period subsequent to photo-charge (i.e., no external current applied to the EDLC section) was found to be roughly the same as that obtained without this resting period, demonstrating the excellent capacity retention (i.e., low self-discharge) of the EDLC section. The photovoltaic module in the above-mentioned work (Scalia et al., 2018) was composed of 4 serially-connected DSSCs as in this present work.

Regarding EDLCs, solid-state polymer electrolytes were recently proposed as a valuable alternative to liquid electrolytes because reliability, safety and bending features are mandatory for miniaturized and smart applications. Moreover, unusual operating conditions may lead liquid electrolytes-based EDLCs to overpressure inside the device, which can sporadically turn in to cell explosion. Solid-state polymer electrolytes have been proposed also to overcome this risk (Fic et al., 2018). Furthermore, the encapsulation of the IL electrolyte into a polymeric matrix would represent a further step toward a leak-less ELDC with the twofold advantage of: (i) reducing the overall IL content and (ii) avoiding the need of additional packaging (Ayalneh Tiruye et al., 2015).

Moreover, with respect to literature(Scalia et al., 2018), employing a polymer as electrolyte yield the subsequent sealing with a specific sealant unnecessary.

**Figure 1:**
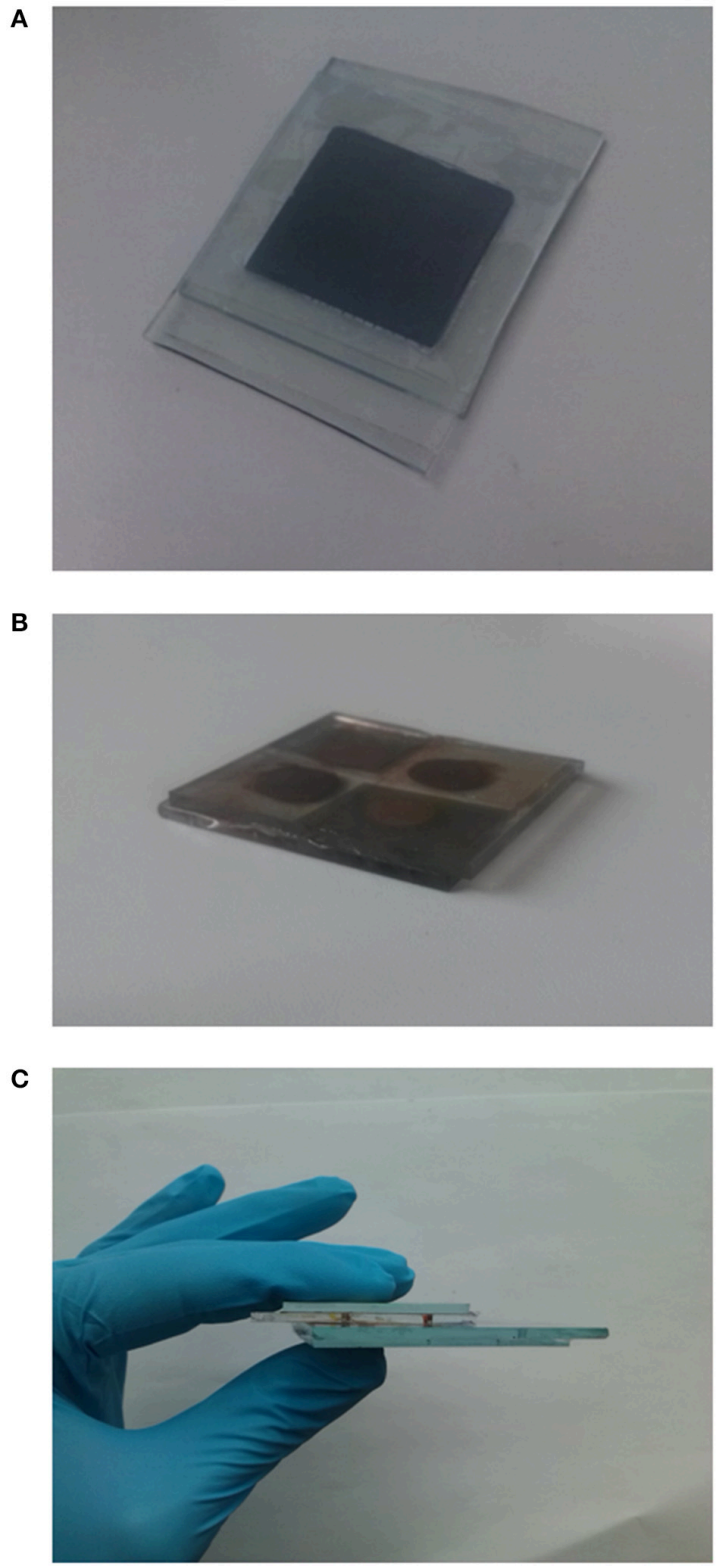
**(A)** Pictures of the polymer electrolyte EDLC, **(B)** Picture of the DSSM section, **(C)** integrated HS device front view.

To the best of our knowledge we present the integration of the aforementioned dye-sensitized solar module (DSSM) and an all-solid-state EDLC for the first time (Figure 1). The two devices are first tested separately while their integration is examined afterwards. The DSSM provides an open circuit voltage of 2.57 V. Thus, the final voltage of the EDLC during photo-charge approached this value. Both the DSSM and the EDLC are fabricated onto glass substrates, paving also the way to a possible future integration in window facades.

## Materials and methods

### Materials

Activated carbon (AC, P4) was provided by SGL Carbon (Germany). Sodium carboxymethyl cellulose (CMC, Walocell CRT 2000) was purchased from Dow Wolff Cellulosics. Conductive carbon (CC, Super C65) was purchased from Imerys Graphite & Carbon. The ionic liquid Pyr_14_TFSI (99.9%, Solvionic) was dried under high vacuum (10^−7^ mbar) for 24 h at 120°C. Its water content after drying, determined by Karl Fischer titration (Mettler Toledo), was below 10 ppm. Benzophenone (for synthesis, ≥99.0%, Merck) was dried under vacuum for 48 h at 40°C. Poly(ethylene oxide) (PEO, Mv = 100,000, Dow Chemical) was dried under vacuum for 48 h at 50°C. The sensitizing dye, cis-bis(isothiocyanato)(2,2'-bipyridyl-4,4'-dicarboxylato)(4,4'-di-nonyl-2'bipyridyl) ruthenium (II) (Z907, Ruthenizer 520-DN) was purchased from Solaronix. TiO_2_ Paste DSL 18NR-AO was purchased from Dyesol. The Meltonix 1170-60 (60 μm) sealant was purchased from Solaronix. Solar cells electrolyte components, sodium iodide (NaI) and iodine (I_2_) were purchased from Sigma-Aldrich (Milan, Italy). 4-tert-butylpyridine (TBP) and methoxypropionitrile (CH_3_OCH_2_CH_2_CN) were purchased from Merck. 7 Ω sq^−1^ sheet resistance fluorine-doped tin oxide (FTO)-coated glass was purchased by Solaronix.

### All-solid-state EDLC fabrication

FTO-coated glass was at first cut in 7.5 × 6 cm^2^ plates by means of a diamond glass-cutter. These plates were then used as substrates and current collector for the EDLC electrodes. Before depositing the active material slurry, these FTO glasses were rinsed with acetone and ethanol in an ultrasonic bath for 10 min to clean the surface. The slurry was prepared by dispersing the electrode components (100 mg of dry mass) in 5 mL ultrapure (milliQ) H_2_O with the appropriate ratio. The composition of the dry electrodes was: 90 wt% of activated carbon (AC), 5 wt% of conductive carbon and 5 wt% binder (CMC). The slurry was then deposited onto the conductive side glass substrate coating a 4 × 4.5 cm^2^ deposition area by means of the doctor blade technique. Symmetrical mass loadings were chosen for the two electrodes. Finally, the coated electrodes were pre-dried overnight at 80°C and then under vacuum conditions for 12 h at 130°C.

The polymer electrolyte, PEO:Pyr_14_TFSI (2:1 mol/mol) + 5 wt% benzophenone (the latter percentage is relative to the weight of PEO), was prepared as reported in Sharova et al. (2016).

Benzophenone was first dissolved in Pyr14TFSI. Then, PEO was hand-mixed together until the formation of a solid material. Afterwards, the electrolyte was vacuum sealed in a pouch bag and annealed for 12 h at 100°C. Subsequently, small electrolyte pieces were sandwiched between two Mylar foils (PPI) and hot-pressed (Servitec Polystat 200T) at 100°C to obtain films of thickness below 100 μm. The electrolyte was then cross-linked in a UV chamber (Uvacube 100) for 6 min (Kim et al., 2010). Finally, the polymer electrolyte was sandwiched between the two glass-supported electrodes and pressed by means of a hot press. The preparation of the electrolyte as the assembly of the device was carried out in dry room (dew point < −60°C).

### Dye-sensitized solar module fabrication

FTO glass plates were used as DSSM substrates. The forward plate was a 4 × 4 cm^2^ FTO glass plate while the backward plate was cut into a 4 × 5 cm^2^ rectangular shape, in order to have additional space for the electrodes' contacts. Four holes (1 mm diameter) were drilled in the backward plate, one for every DSSC, for electrolyte filling. The conductive FTO layers were appropriately patterned with a diamond tip in order to electrically separate a photo-anode from the cathode of a contiguous solar cell. Glass plates were rinsed in acetone and ethanol in an ultrasonic bath for 10 min. Afterwards, dissimilar Pt layers were deposited onto the cathode regions of the forward and backward plates of the glass substrates, respectively, 3 and 10 nm thick, by means of sputtering technique (EM ACE 600, LEICA). Sputtering parameters were set to 30 mA current and 0.04 nm/s deposition rate. Of course, different deposition times were adopted for the front or back irradiated cells. In order to deposit the semiconductor paste layer, adhesive tapes (80 μm thick) with circular holes were placed on the glass plates. For the front side and backside irradiated cells the hole diameters were set to 10 and 12 mm respectively. The paste was deposited onto the adhesive mask by means of the doctor blade technique. The FTO-glasses with TiO_2_ paste were calcined at a temperature of 510°C for 30 min. The sintered electrodes were then soaked for 15 h in the 0.3 mM Z907 solution in ethanol. The devices were sealed by means of a hot press utilizing 2 × 60-μm-thick Meltonix films. The temperature of the hot press was set to 120°C. The liquid electrolyte was inserted by vacuum backfilling through the 4 holes previously drilled. Electrolyte composition was a 0.45 M sodium iodide, 0.056 M iodine and 0.55 M 4-tert-butylpyridine dissolved in methoxypropionitrile (MPN).

### Integration of the DSSM and all-solid-state EDLC

DSSM and EDLC were integrated in a single structure by means of a hot press. Two Meltonix films (each 60 μm thick) were placed between the DSSM and the EDLC cathode, leaving the DSSM contacts region outside the EDLC surface in order to easily connect harvesting and storage contacts during photocharge. With this method also a proper sealing of the backward DSSM holes was ensured.

### Photo-electrochemical characterization

For the evaluation of the DSSM performance a HelioSim-CL60 (Voss Electronics) solar simulator and a PG510/590 (Heka) potentiostat were used in order to measure the photo-generated current under simulated solar radiation with an AM1.5 spectrum. During this phase, the potenstiostat terminals were connected to the PV section electrodes of the HS device. The EDLC performance was evaluated by means of galvanostatic constant current (GCC) cycling and cyclic voltammetry (CV) executed on a programmable multi-channel potentiostat-galvanostat (VMP3, Biologic Science Instruments, France). The tests were performed at 20°C in a climatic chamber (Binder, KBF-115). Electrochemical impedance spectra (EIS) was recorded at Voc, by means of an Impedance/Gain-Phase Analyzer 1260 (Solartron Analytical) between 0.1 MHz and 1 mHz, with an AC amplitude of 10 mV. Upon photo-charge, the voltage of the EDLC was measured with a potentiostat PG 510/590 (Heka), while the HS device was irradiated by the solar simulator.

## Results and discussion

**Figure 2:**
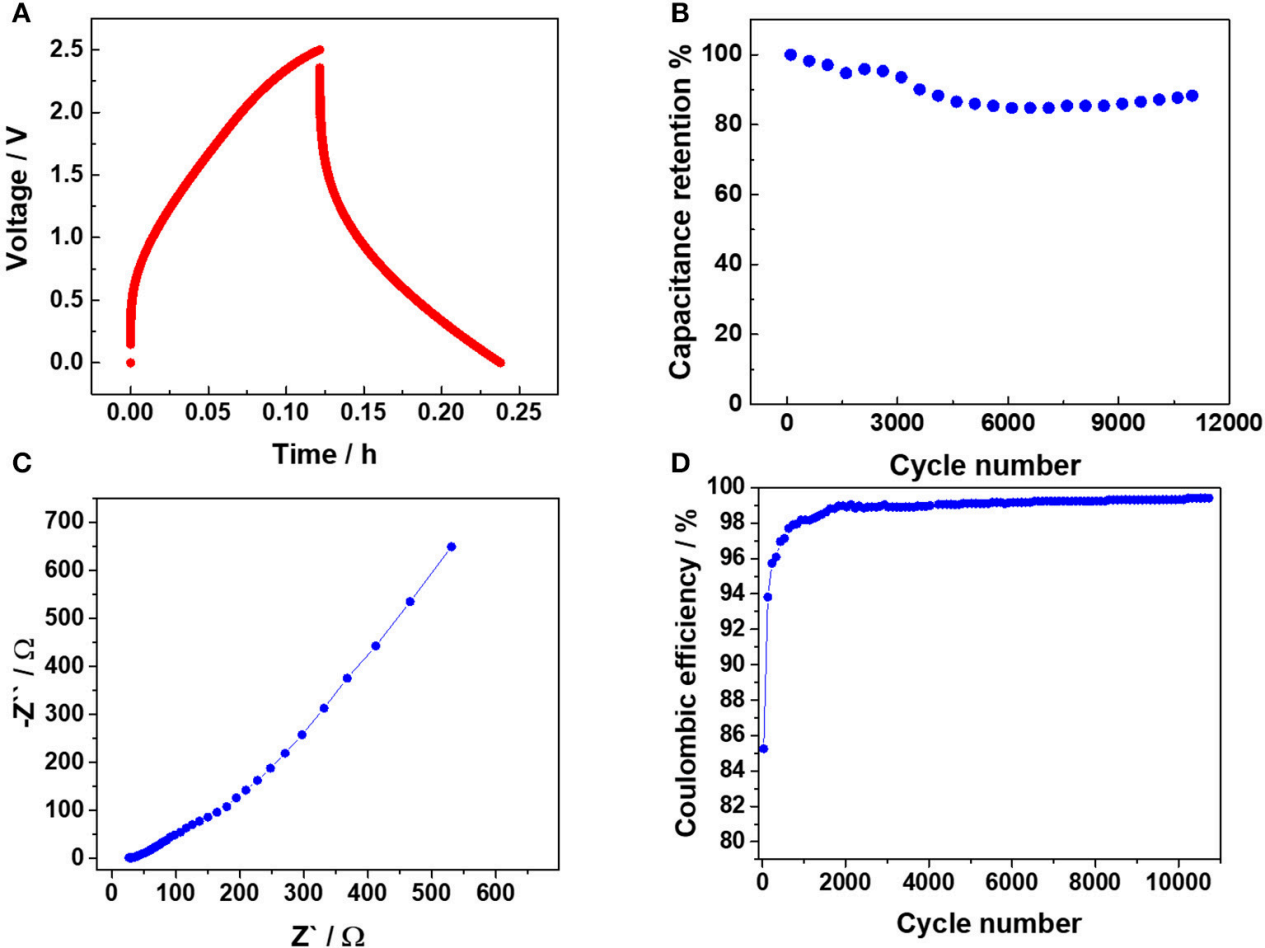
Electrochemical performance of the all-solid-state capacitor employing a PEO-Pyr_14_TFSI polymer electrolyte. **(A)** Typical voltage profile recorded for charge/discharge at 2 mA (0.11 mA cm^−2^). **(B)** Capacitance retention test performed at 20 mA (1.11 mA cm^−2^), **(C)** EIS spectrum (Nyquist plot) collected between 1 mHz and 1 MHz with a 10 mV amplitude, **(D)** Cycling stability test (coulombic efficiency) performed at 20 mA (1.11 mA cm^−2^) for 11,000 cycles.

The electrochemical performance of the all-solid-state EDLC is summarized in Figure 2. Figure 2A shows a typical galvanostatic charge-discharge (GCD) voltage profile for an imposed current of 2 mA (0.11 mA cm^−2^). A deviation from the ideal capacitive response can be clearly noticed due to the poor conductivity of the FTO glass conductive substrate, but also the moderate ionic conductivity of the PEO-Pyr_14_TFSI electrolyte in comparison with the previously used neat IL (Scalia et al., 2018). Since the charging and discharging behavior is non-linear, the average capacitance (*C*_A_) was evaluated directly from the SC discharging energy, via the following formula:

(1) $$\begin{array}{r} \frac{1}{2}\;C_{A}V_{M}^{2}=i\;\int_{t_{i.d}}^{t_{f.d}}V\;dt=E_{EDLC} \end{array}$$

Here *V*_M_ is the maximum voltage reached during the GCD (2.5 V), *V* is the EDLC voltage, *i* is the imposed discharge constant current, *t*_i.d_ is the initial discharge time, *t*_f.d_ is the final discharge time, and *E*_EDLC_ is the EDLC energy evaluated during discharge phase. The *C*_A_ value of 0.16 F was calculated, namely providing a normalized capacitance of 9 mF·cm^−2^. Impressively, such a polymer electrolyte-based EDLC offers a capacitance value almost four times higher than that of the liquid (organic) electrolyte EDLC proposed by Chien et al. (2015), which is one of the very few examples of photo-capacitor with a PV section composed by a module (series connected cells) instead of a single solar cell. This further demonstrates the highly remarkable performance of the storage device developed in this work. In addition, the polymer electrolyte-based EDLC does not show sign of electrolyte degradation, which are indeed well evidenced by the voltage plateau observed at about 2 V upon GCD measurement in the work of Chien et al. (Chien et al., 2015). It testifies that the all-solid-state glass-based EDLC possesses a stability in the chosen voltage window, difficult to find in literature for high voltage PV harvester-EDLC integrated devices. Such a high stability is rather important performance, since a huge number of photo-charge discharge cycles are to be expected for this kind of harvesting-storage devices. Figure 2B shows the capacitance retention of the EDLC section. More than 10,000 GCD cycles were performed at an imposed current of 20 mA (1.11 mA cm^−2^). After an initial phase (3,000 cycles) where a small decrease is observed, the capacitance stabilizes at almost 90% of the initial value. After 5,000 cycles the capacity slowly increased indicating that the EDLC section posseses outstanding reliability upon long-term cycling.

Electrochemical impedance spectroscopy (EIS) of the PEO-Pyr_14_TFSI-based EDLC was performed, which results are reported in the Nyquist plot of Figure 2C. As suggested by Zhang et al., (Zhang and Pan, 2015) the equivalent series resistance (ESR) was evaluated by the real part of the impedance at 1 kHz, yielding a value of 33.8 Ω. Almost the same value was found, evaluating the ESR by the voltage drop of the CD reported in Figure 2A (36 Ω). Even if a consistent part of the ESR is ascribable to the FTO resistance, the increase of the ESR with respect to our previous work (Scalia et al., 2018) featuring pure IL (26.8 Ω), suggests that the polymer electrolyte provides an important contribution to the overall ESR. In the intermediate frequency region, a linear behavior with a slightly lower slope with respect to the usual 45° Warburg trend is observable. This region can be ascribed to the limited ion diffusion in the electrodes pore structure, once more associated to the use of the all-solid-state electrolyte. Finally, the values at low frequencies are not parallel to the imaginary axis, meaning that the response of the SC deviates from that of an ideal capacitor in agreement with the voltage profile upon GCD (see Figure 2A). Again, the limited conductivity of the polymer electrolyte certainly plays a relevant role here. Figure 2D shows the coulombic efficiency related to the long-term stability test (see Figure 2B) of the all-solid-state EDLC via GCD at 20 mA (1.11 mA cm^−2^). After the initial activation the coulombic efficiency was always higher than 99%, showing relevant electrical double layer capacitance features.

**Figure 3:**
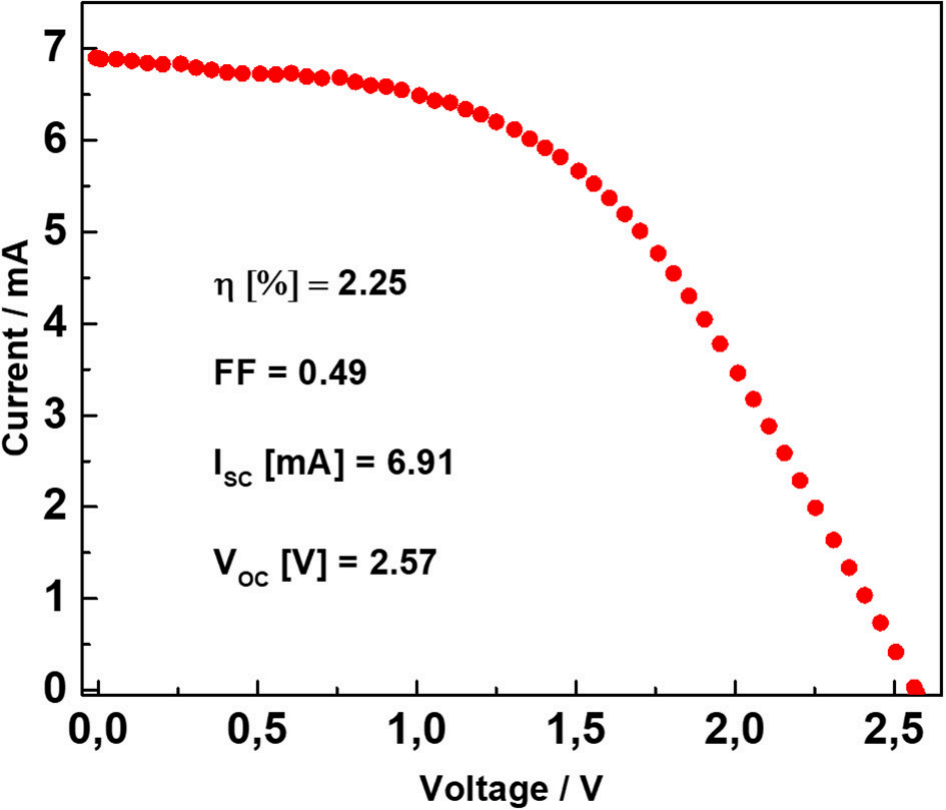
Current–voltage curve of the DSSM performed under 1 sun illumination condition (100 mW cm^−2^). In the inset, the photovoltaic peculiar parameters of the DSSM are reported.

Figure 3 shows the current-voltage characteristic of the DSSM. It was obtained under 1 sun (1,000 Wm^−2^) illumination conditions. In the inset the most relevant photovoltaic parameters (photovoltaic efficiency, fill factor, short circuit current, and open circuit voltage) are reported. Additional information regarding the DSSMs herein investigated are available in our previous work (Scalia et al., 2018).

**Figure 4:**
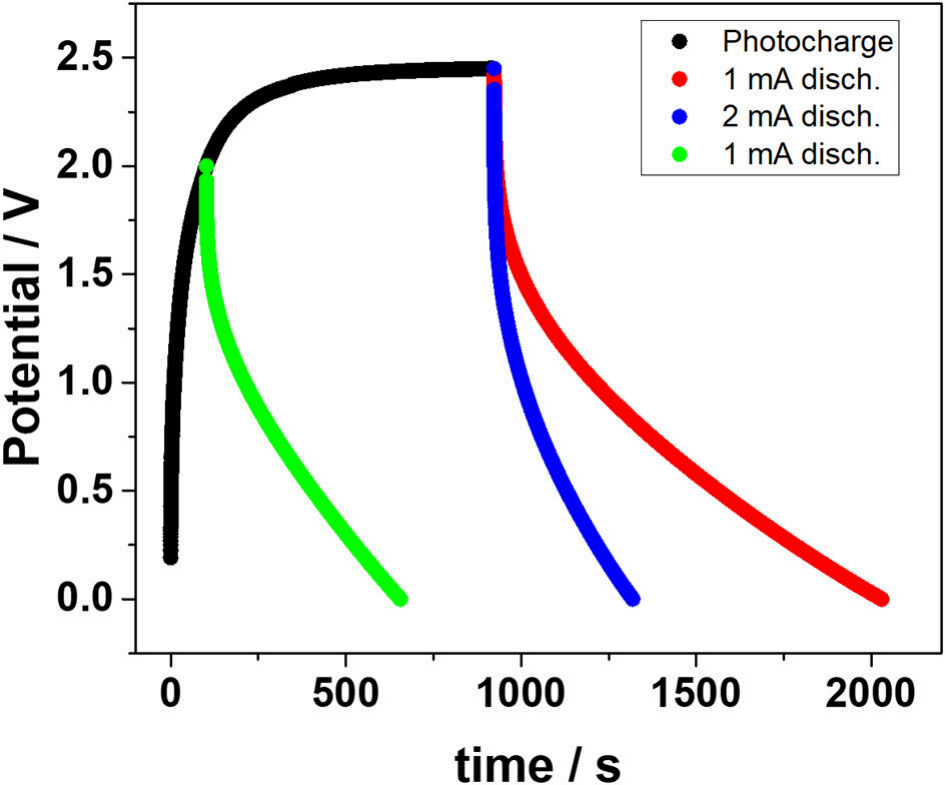
Storage performance of the HS device. The photo-charge was performed under 1 sun radiation conditions while the discharge curves were recorded under constant current conditions.

Figure 4 displays the photo-charge and subsequent discharge curves of the integrated HS device. In the first part of the photo-charge the EDLC voltage rapidly rises until about 2 V and then evolves into a plateau above 2.3 V. This trend is due to the EDLC voltage approaching the open circuit voltage of the DSSM, resulting in a lower current supplied to the EDLC. Nonetheless, the photo-charge was limited to 2.45 V for practical reasons. Upon discharge, two different negative current values were applied, namely 1 and 2 mA (0.056 and 0.11 mA·cm^−2^). A remarkable capacity value over 0.017 mAh·cm^−2^ was found for the imposed discharge current of 1 mA. Although lower than that obtained for the EDLC employing the neat IL electrolyte (Scalia et al., 2018), this value is still higher than the majority of liquid-based electrolyte storage section photo-capacitors reported in the literature (Xu et al., 2014, 2016; Chien et al., 2015).

The galvanostatic discharge after limiting the photo-charge to 2 V was also studied in order to probe the performance of the HS device upon shorter photo-charging time. This experiment is highly significant for real HS applications where rather short photo-charging time maybe available to cope with subsequent user requests.

The overall photon to electrical conversion and storage efficiency (OPECSE) was evaluated with the following formula: (Xu et al., 2014, 2016; Kim et al., 2017)

(2) $$\begin{array}{r} \eta \;\left(OPECSE\right)=\frac{\frac{1}{2}\;C_{A}\;V^{2}}{G\;t\;S} \end{array}$$

Here *C*_A_ (0.16 F) is the average capacitance evaluated from *E*_*EDLC*_ during the discharge phase, *V* is the EDLC voltage, *G* is the impinging electromagnetic density radiation (100 mW·cm^−2^), *t* is the photo-charge time and S is the active surface area of the DSSM.

**Figure 5:**
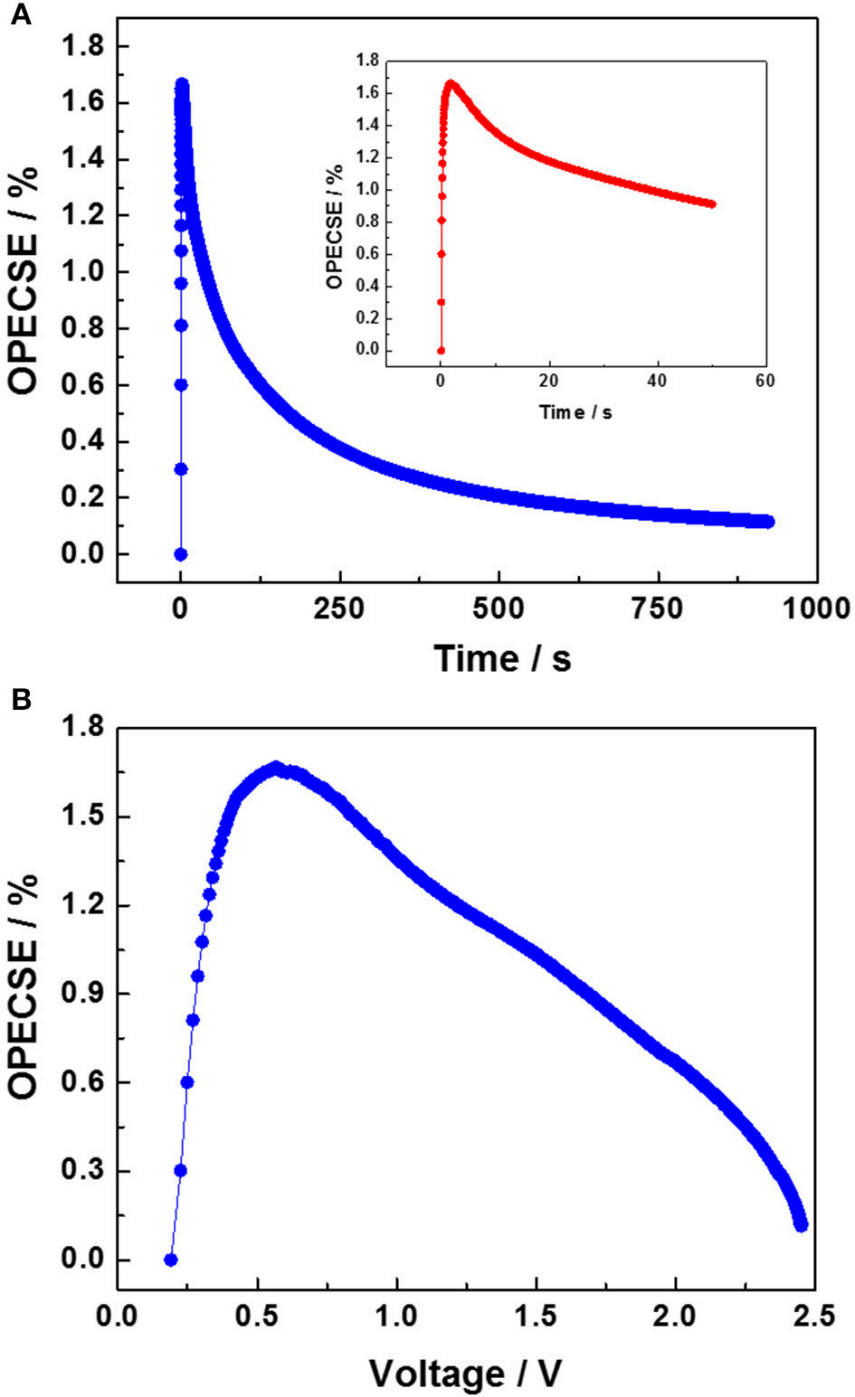
**(A)** OPECSE values plotted as a function of the photo-charging time (in the inset, the values corresponding to the first 50 s of photo-charge are plotted), **(B)** OPECSE values plotted as a function of the EDLC voltage.

Figure 5A shows the OPECSE value as a function of the photo-charging time. The obtained profile is in accordance with the literature and presents a maximum of 1.67% in the very first part of the photo-charge when the voltage varies linearly with time. Then, the OPECSE gradually decreased since the provided incoming electromagnetic energy was kept constant while the EDLC voltage asymptotically approached the DSSM V_OC_, limiting the energy stored in the EDLC section. The inset in Figure 5A shows the OPECSE variation during the initial 50 s of photo-charge. Interestingly, for this charging step, in which the EDLC reaches a voltage of 1.7 V, a still remarkable efficiency corresponding to 60% of the maximum OPECSE is achieved. Figure 5B shows the OPECSE as a function of the EDLC voltage reached during photo-charge. This analysis is crucial since, as stated before, the voltage output of an integrated device needs to be higher than 1 V in order to drive electronic utilizers. After reaching the maximum value around 0.6 V, the OPECSE decreases linearly. Ideally, the maximum OPECSE should be reached at a voltage higher than 1 V and/or its value should remain constant up to voltages close to the DSSM's *V*_OC_. Nevertheless, the almost linear decrease (rather than an abrupt decrease) of the OPECSE vs. increasing voltages is still considered to be a good compromise. Additionally, the number of the cells in the DSSM can be adjusted to optimize the OPECSE to the utilizers' requirements.

Overall, further improvement could be foreseen in a future work. The EDLC section could probably bear a higher voltage, given the stability of the ionic liquid up to 3.5 V (Varzi et al., 2014). Thus, a DSSM with 5 serially connected cells (expected 3.1 V) could more exploit better the whole potential of the ELDC. However, as it was designed in this work, the DSSM required an even number of cells. If employing 6 cells, the open circuit voltage of the DSSM would instead exceed stability window of the electrolyte.

## Conclusions

In summary, here we have reported a novel high voltage photo-capacitor composed by a 4 serially connected cells into a DSSM and an all-solid-state EDLC. The system achieved a highly remarkable photo-charging potential of 2.45 V, never obtained before for a HS device including a solid-state electrolyte storage section. The DSSM and EDLC sections were fabricated onto glass substrates and finally integrated into a compact structure. The EDLC storage section employing PEO-Pyr_14_TFSI as electrolyte, allowed long photo-charge/discharge cycling life. Additionally, the use of the solid-state electrolyte facilitates the sealing of the whole structure, still providing appealing performance above the state-of-the-art reported in the literature. A remarkable capacity value over 0.017 mAh·cm^−2^ was achieved upon discharging the cell at 1 mA after photo-charge, proving the suitable characteristics of the HS for applications of practical interest.

A further increment of PV efficiency and, as a consequence, of the OPECSE could be foreseen by tuning the electrolyte layer thickness or by designing a Z-type DSSM. This would render this type of HS device a really competitive technology able to satisfy a variety of energy storage applications.

## Author contributions

AS and AV designed the experimental work. AS performed the experimental work. All authors analyzed the results and contributed to write the manuscript.

### Conflict of interest statement

The authors declare that the research was conducted in the absence of any commercial or financial relationships that could be construed as a potential conflict of interest.
